# Shone’s complex and aortic dissection: case report and review of a rare, underdiagnosed congenital heart disease

**DOI:** 10.1186/s13019-022-01768-z

**Published:** 2022-02-23

**Authors:** Steven Sinfield, Sachini Ranasinghe, Stephani Wang, Fernando Mendoza, Ali Khoynezhad

**Affiliations:** 1 Department of Medicine, University of California, Irvine, 333 The City Blvd. West, Suite 400, Orange, CA 92868 USA; 2 Department of Medicine, Division of Cardiology, University of California, Irvine, Irvine, USA; 3 Memorial Care Heart and Vascular Institute, MemorialCare Long Beach Medical Center, Long Beach, USA

**Keywords:** Shone’s syndrome, Shone’s complex, Congenital heart disease, Aortic dissection, Bentall procedure, Aortic aneurysm, LVOT obstruction, Patient prosthesis mismatch, MAZE procedure

## Abstract

**Background:**

Shone’s complex is a rare congenital heart disease consisting of a variety of left ventricular inflow and outflow tract lesions. Patients typically present in childhood requiring early surgical intervention; however, with improved surgical techniques, these patients are surviving later into adulthood. This increased survival comes with a new set of medical complications that providers need to be aware of.

**Case presentation:**

A 27 year old man with a complex cardiac history including an incomplete Shone’s complex and persistent symptomatic atrial flutter presented with sharp chest pain radiating to his back. He was found to have type A aortic dissection on imaging in the setting of severe patient-prosthesis mismatch. He had multiple valvular surgeries in childhood. The patient was being followed-up as an outpatient for an enlarging chronic aortic aneurysm and was non-compliant with his medications. He was taken emergently to the operating room for a skirted Bentall procedure, aortic valve replacement, and right sided MAZE.

**Conclusions:**

Shone’s complex is a rare congenital heart disease associated with significant morbidities including atrial flutter, patient-prosthesis mismatch, and aortic dissection. As patients continue to live longer into adulthood with this disease, it is important to raise awareness of this rare syndrome for providers and highlight its potential complications. Further research is needed to determine appropriate guidelines for when to intervene on aortopathy-associated CHD.

## Background

Shone’s complex (SC) or syndrome is a rare congenital heart disease (CHD) that consists of 4 main anomalies: coarctation of the aorta (CoA), subaortic stenosis, parachute mitral valve (PMV) and supravalvular mitral ring. Complete SC consists of all 4 abnormalities, while incomplete SC, which is much more common, consists of 2–3 anomalies [1]. Most case series now define SC as a combination of mitral valve disease plus at least one level of left heart obstruction. Typical mitral stenosis is most common, with some case series showing it to be 70–90% of all SC cases [2, 3].

The pathogenesis of SC is hypothesized to initially be caused by a disruption to the left ventricular inflow tract (LVIT) from a congenital mitral valve anomaly during embryogenesis, leading to underdevelopment of the left ventricle (LV) cavity [3, 4]. Although previously a disease of unknown cause, a recent study found that 11% of SC cases were associated with MYH6 de novo mutations, which encodes for the cardiac alpha-myosin heavy chain that is highly expressed in the embryonic heart [5].

Patients typically present in childhood with a spectrum of symptoms including poor feeding, failure to thrive, and signs of reduced cardiac output [6]. Management involves cardiac surgery during childhood and the most common interventions include surgical coarctation repair, subaortic resection, and mitral valve repair [2]. Most published studies on SC come from the pediatric literature, likely due to the rarity of this disease as an adult. However, with improved surgical techniques over the last 40 years, there has been a 24% increase in adults living with CHD, including SC patients [7]. As patients with SC continue to live longer, this will come with a new set of medical complications in adulthood that providers need to be aware of.

This is a case of a young adult patient diagnosed with incomplete SC during childhood who presented with persistent symptomatic atrial flutter and an acute type A dissection in the setting of a growing proximal aortic aneurysm. This case will highlight a rare form of CHD and the potential complications of SC management in adult patients.

## Case presentation

**Fig. 1:**
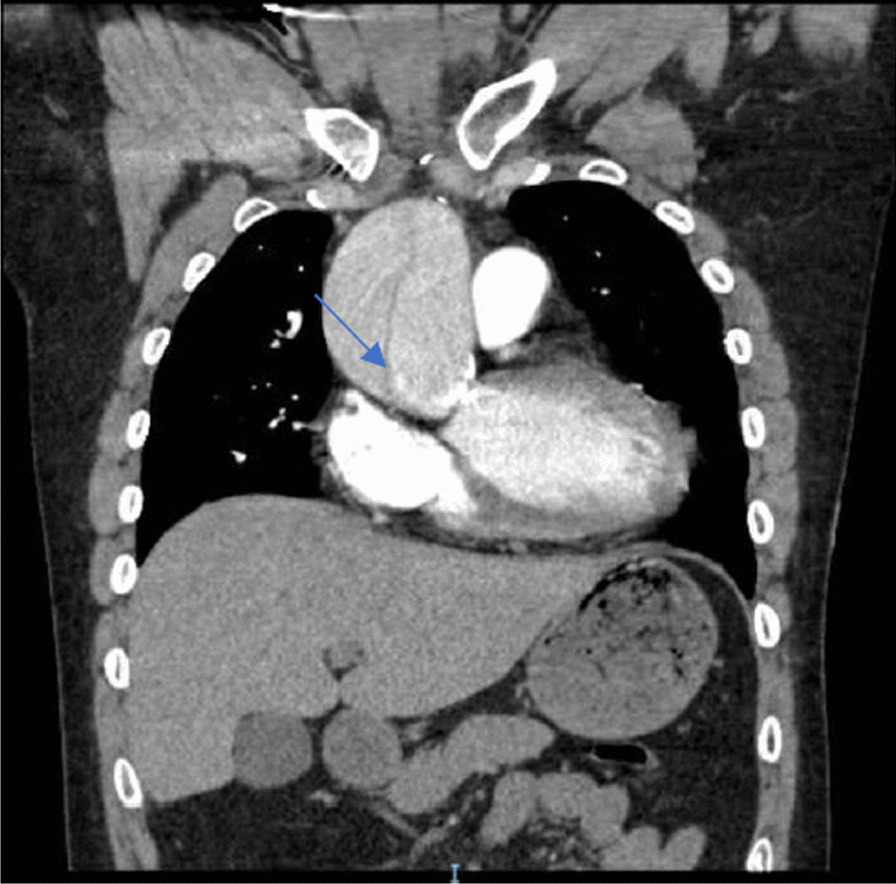
CTA chest coronal view demonstrating acute aortic dissection in setting of chronic aortic dilation (blue arrow)

A 27 year old male with a past medical history significant for incomplete SC and persistent atrial flutter presented to an outside hospital with 10/10 intensity, sharp chest pain radiating to his back after having a bowel movement. Computed tomography angiography (CTA) of the aorta demonstrated an acute type A aortic dissection (AD) (Fig. 1) and he was transferred to our institution for higher level of care. Upon arrival, the patient was afebrile, had a systolic blood pressure in the low 90’s, and was saturating 95% on room air. He was in atrial flutter with a heart rate in the 70’s. He was started on an esmolol and nicardipine drip as needed.

He has a complex congenital cardiac history of a shone complex variant including coarctation of the aorta (CoA), subaortic stenosis, pulmonary stenosis, atrial septal defect (ASD), ventricular septal defect (VSD), and mitral stenosis. During his first days of life, he underwent a pulmonary valvotomy, LV apical to descending aortic conduit, and ASD/VSD closure. At 1 year of life, he underwent a Konno procedure with a 17 mm mechanical St Jude aortic valve replacement and mechanical 23 mm St Jude mitral valve replacement. However, as a full grown adult, both of his valves were now consistent with severe patient-prosthesis mismatch. His most recent transthoracic echocardiogram (TTE) one year prior demonstrated borderline mechanical valve aortic stenosis with a mean gradient of 23 mm Hg, severe mechanical mitral valve stenosis with an inflow gradient of 11–12 mm Hg, a normal functioning pulmonic valve with no evidence of regurgitation, and indeterminate diastolic dysfunction.

**Fig. 2:**
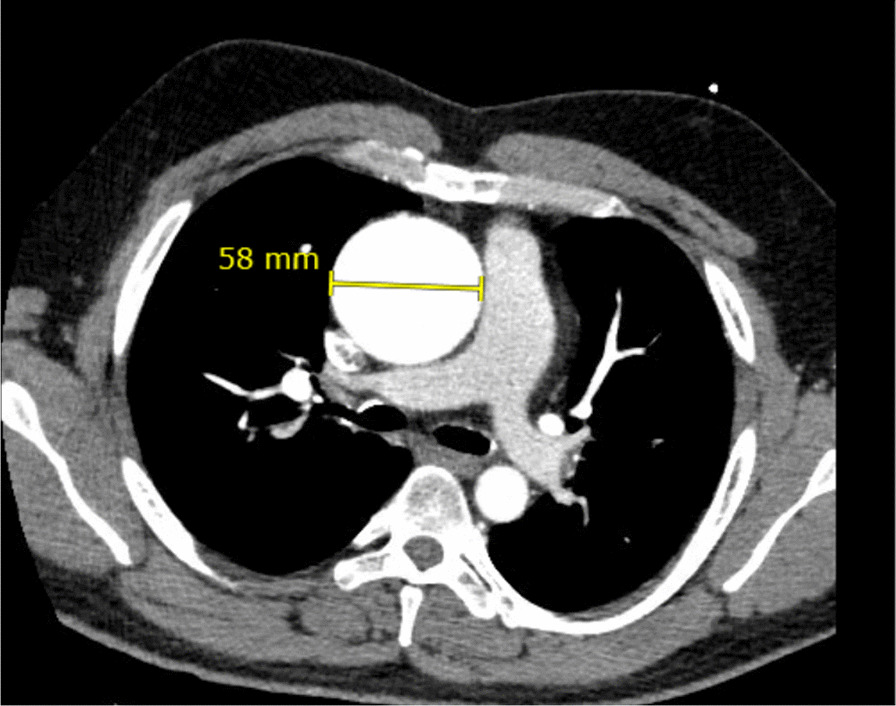
CTA chest axial cut demonstrating stable fusiform aneurysmal dilation of ascending thoracic aorta measuring 5.8 cm in maximum transverse diameter, mildly increased from previous CT 4 mo prior. No evidence of aortic dissection or hematoma

Over the last few years, the patient underwent annual monitoring for an enlarging aortic aneurysm, initially noted to be 5.3 cm in 2016. Despite extensive counseling, the patient continued to be noncompliant with his beta blocker and participated in heavy weightlifting placing increased stress on his aortic wall. He had yearly scans and his most recent CTA aorta in 2020 demonstrated growth of the thoracic aortic aneurysm to 5.8 cm with no evidence of dissection or hematoma (Fig. 2). This scan was done less than one month prior to his hospital presentation, and he was scheduled for follow up with his congenital cardiologist to discuss surgical options which he never made it to.

**Fig. 3:**
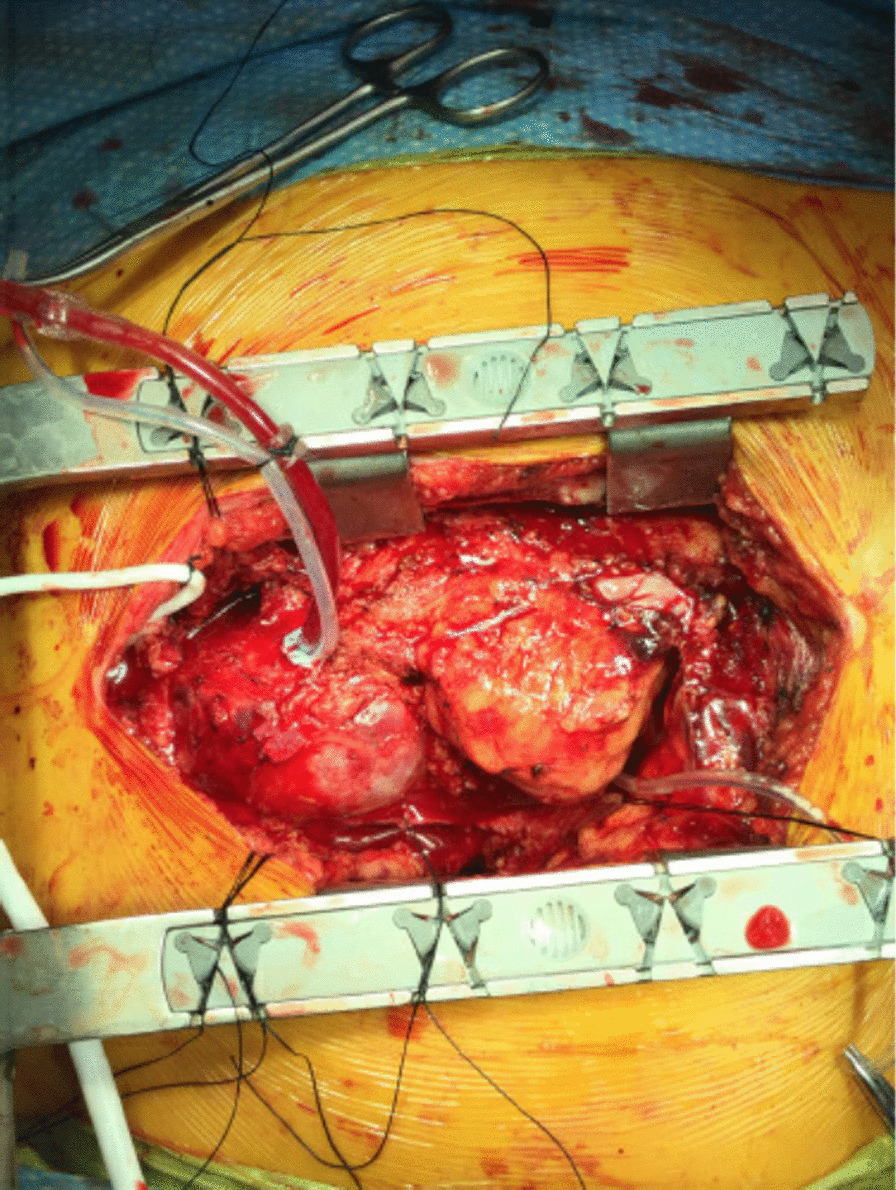
OR view of re-do sternotomy with large aortic root aneurysm and acute type A aortic dissection

On presentation, the patient was alert and oriented with no motor/sensory deficits. He was transferred to the CCU and scheduled for emergent redo sternotomy. In the operating room, a re-do sternotomy was performed through the same skin incision. There were severe adhesions leading to nearly 2 h of dissection and an unintended diaphragmatic RV injury requiring a definitive pericardial patch for repair. The patient was noted to have a large aneurysmal aortic root from the ascending aorta up to the innominate aorta (Fig. 3). The ascending aorta was completely resected and a hemiarch replacement was performed using a 32 mm branched vessel Gelweave Ante-Flo graft (copyright Terunmo, Sunrise, Fl). Next, the mechanical aortic valve was explanted and the surrounding aneurysmal tissue was resected. Replacement with a larger valve was mandatory given the severe aortic valve patient-prosthesis mismatch. However, the aortic annulus was severely calcified, due to the previous Konno procedure, making a root enlargement impossible. Therefore, instead of standard Bentall (aortic root replacement), a skirted Bentall operation was performed by moving the aortic annulus about 8 mm into the aorta. This allowed for a 23 mm St Jude’s mechanical Regent valve (Copyright Abbott, Chicago, IL) to be utilized in a 26 mm Valsalva graft (copyright Terunmo, Sunrise, FL). The aortic root calcifications also involved the left and right main coronary arteries, making coronary mobilization hazardous. Therefore, the Cabrol procedure was performed, suturing an 8 mm graft to the left and right main coronary orifice with a side-to-side anastomosis to the Valsalva graft.

Finally, a CryoMaze probe (copyright Atricure, Mason, OH) was used to perform a right sided MAZE for his persistent atrial flutter. This was done by performing a superior to inferior vena cava lesion, connecteing a lesion to the right atrial appendage and to the annulus of the tricuspid valve using cryothermia. A decision was made not to replace the mechanical mitral valve as the patient lacked symptoms of shortness of breath prior to the AD and the already prolonged, complex operation he needed.

The patient did well post-operatively with no major complications. He required low dose epinephrine drip for right ventricular support and dobutamine drip for inotropy for two days. He remained in normal sinus rhythm on continuous cardiac monitoring throughout his recovery and EKG on day of discharge showed normal sinus rhythm. At three and six month follow ups, the patient remained asymptomatic. His most recent TTE findings demonstrated a normal functioning aortic valve with effective orifice area of 1.5 cm and a mean gradient of 15 mm Hg.

## Discussion

Shone’s complex is a rare form of CHD, comprising about 0.6% of total cases [6]. For patients diagnosed during childhood, management involves early surgical repair with 63% of patients requiring multiple interventions [8]. The variability of lesions seen in incomplete SC makes the formulation of a generalized surgical strategy problematic. Two retrospective studies analyzing surgical management of SC recommend left ventricular outflow tract (LVOT) obstruction be addressed with initial surgery, while valve repair should be done whenever possible [6, 8, 9].

The rarity of this disease presentation has led to a deficit of published literature on SC in adult patients. This has thus created a decreased awareness of the syndrome, leading to concern that SC may be under-recognized in the adult population. There have been multiple case series and reports of patients who were initially diagnosed with isolated CoA or other LVOT obstructions who later presented with progressive mitral valvular disease in adulthood and were found to have undiagnosed SC [4, 10, 11]. A retrospective study by Aslam et al., found that only 39% of SC cases were diagnosed in childhood and that the mean age of diagnosis was 32 years old [3].

With the improvement of CHD surgical techniques, patients with CHD, specifically SC, are living much longer into adulthood. Mortality is generally limited to infancy and case series have shown favorable outcomes: on average SC patients can go 24 years without clinical events and that up to 90% of patients are transplant free by 30 years old [3, 10]. However, due to their complex anatomy and multitude of cardiac surgeries, SC patients have a significantly higher health services utilization compared to the general population. Nearly 50% of SC patients are hospitalized as adults, most commonly for arrythmias, heart failure, and new surgical interventions [3]. This increased morbidity in adulthood may be associated with significant hemodynamic changes from left sided obstructive lesions as studies have shown that SC patients have significant LV remodeling and diastolic dysfunction in adulthood [12].

Our patient in this case presented with acute aortic dissection in the setting of a slow growing proximal aortic aneurysm treated with a skirted Bentall procedure and aortic valve replacement. To our knowledge, there has only been one other case report of a patient with incomplete SC presenting with a type A dissection years after a CoA repair [11]. SC patients with AVR during their early life are at high risk for complications. While there are no guideline recommendations for prophylactic surgery specifically for aortopathy-associated CHD in adults, the current STS, AATS, and ACC/AHA guidelines all recommend prophylactic replacement of the aorta once it reaches 5.5 cm [13]. Our patient should have been referred for surgical consult and prophylactic surgery for aortic dilation once the aorta surpassed 5 cm, rather than waiting until the aorta was greater than 5.5 cm.

Another major complication for CHD patients in adulthood is patient-prosthesis mismatch (PPM), which possibly contributed to further dilation of the aortic aneurysm in this patient. PPM occurs when the area of a functioning prosthetic valve is too small for the body surface area, due to normal somatic growth of cardiac tissue [14]. In our case, the patient had both aortic and mitral valve replaced in childhood, which both became too small for his size. PPM is determined by taking the ratio of the average effective orifice area of the valve to the patient’s body surface area. The indexed effective orifice area for this patient’s aortic mechanical valve was 0.58 cm^2^/m^2^ and for his mitral mechanical valve was 0.52 cm^2^/m^2^ indicating severe PPM of both valves [14]. Transesophageal echocardiography done preoperatively demonstrated borderline aortic stenosis with a mean gradient of 23 mm Hg and max velocity of 2.89 m/s and moderate-severe mitral stenosis with a mean gradient of 10 mm Hg. It has been shown that severe PPM is associated with a two-fold increase in mortality, worsening functional capacity and higher rates of HF hospitalizations in CHD patients [14]. Nearly one third of CHD patients require interventions in adulthood mainly for degeneration of bioprosthetic heart valves and patient-prosthesis mismatch from somatic growth [3]. It is important for physicians to consider these potential complications in SC patients and monitor appropriately, particularly due to contemporary extended life expectancy.

Finally, atrial flutter in patients with CHD can have debilitating symptoms and substantially increase the risk of thrombus formation, stroke, and tachycardia-induced cardiomyopathy. Right-sided MAZE procedure addresses right sided atrial flutter and provides curative treatment of atrial flutter. In patients with concomitant atrial fibrillation, the atrial fibrillation-free success of bi-atrial MAZE procedure is above 80% at five year follow up [15].

## Conclusion

Shone’s complex is a rare, underrecognized syndrome associated with a relatively low mortality but carries substantial morbidities. Given the increasing number of patients with SC surviving to adulthood, it is important to gain a better understanding of the prognosis and tailor appropriate management. This case should bring increased awareness to SC and its potential complications such as atrial flutter and aortic dissection. Further research is needed to determine appropriate guidelines for when to intervene on aortopathy-associated CHD and reliable markers for dissection-risk in CHD patients.

## Data Availability

Data sharing is not applicable to this case report as no datasets were generated or analyzed in our article.

## Abbreviations

SC: Shone’s complex
CHD: Congenital heart disease
CoA: Coarctation of the aorta
PMV: Parachute mitral valve
MV: Mitral valve
LVIT: Left ventricular inflow tract
LV: Left ventricle
CTA: Computed tomography angiography
AD: Aortic dissection
ASD: Atrial septal defect
VSD: Ventricular septal defect
EKG: Electrocardiogram
PH: Pulmonary hypertension
LVOT: Left ventricular outflow tract
TTE: Transthoracic echocardiography
AVR: Aortic valve replacement
PPM: Patient prosthesis mismatch
BSA: Body surface area
